# Longitudinal Monitoring of DNA Viral Loads in Transplant Patients Using Quantitative Metagenomic Next-Generation Sequencing

**DOI:** 10.3390/pathogens11020236

**Published:** 2022-02-11

**Authors:** Ellen C. Carbo, Anne Russcher, Margriet E. M. Kraakman, Caroline S. de Brouwer, Igor A. Sidorov, Mariet C. W. Feltkamp, Aloys C. M. Kroes, Eric C. J. Claas, Jutte J. C. de Vries

**Affiliations:** Clinical Microbiological Laboratory, Department of Medical Microbiology, Leiden University Medical Center, 2333 ZA Leiden, The Netherlands; e.c.carbo@lumc.nl (E.C.C.); a.russcher@lumc.nl (A.R.); m.e.m.kraakman@lumc.nl (M.E.M.K.); c.s.de_brouwer@lumc.nl (C.S.d.B.); i.sidorov@lumc.nl (I.A.S.); m.c.w.feltkamp@lumc.nl (M.C.W.F.); a.c.m.kroes@lumc.nl (A.C.M.K.); e.c.j.claas@lumc.nl (E.C.J.C.)

**Keywords:** viral metagenomics, pathogen detection, quantification, next-generation sequencing, load monitoring

## Abstract

Introduction: Immunocompromised patients are prone to reactivations and (re-)infections of multiple DNA viruses. Viral load monitoring by single-target quantitative PCRs (qPCR) is the current cornerstone for virus quantification. In this study, a metagenomic next-generation sequencing (mNGS) approach was used for the identification and load monitoring of transplantation-related DNA viruses. Methods: Longitudinal plasma samples from six patients that were qPCR-positive for cytomegalovirus (CMV), Epstein-Barr virus (EBV), BK polyomavirus (BKV), adenovirus (ADV), parvovirus B19 (B19V), and torque teno-virus (TTV) were sequenced using the quantitative metagenomic Galileo Viral Panel Solution (Arc Bio, LLC, Cambridge, MA, USA) reagents and bioinformatics pipeline combination. Qualitative and quantitative performance was analysed with a focus on viral load ranges relevant for clinical decision making. Results: All pathogens identified by qPCR were also identified by mNGS. BKV, CMV, and HHV6B were additionally detected by mNGS, and could be confirmed by qPCR or auxiliary bioinformatic analysis. Viral loads determined by mNGS correlated with the qPCR results, with inter-method differences in viral load per virus ranging from 0.19 log_10_ IU/mL for EBV to 0.90 log_10_ copies/mL for ADV. TTV, analysed by mNGS in a semi-quantitative way, demonstrated a mean difference of 3.0 log_10_ copies/mL. Trends over time in viral load determined by mNGS and qPCR were comparable, and clinical thresholds for initiation of treatment were equally identified by mNGS. Conclusions: The Galileo Viral Panel for quantitative mNGS performed comparably to qPCR concerning detection and viral load determination, within clinically relevant ranges of patient management algorithms.

## 1. Introduction

Opportunistic viral infections frequently occur after solid organ or hematopoietic cell transplantation, with associated morbidity and mortality of up to 40% [1]. Successful prevention and early detection of viral infections including reactivations are the cornerstones of transplant patient management. For effective pre-emptive and therapeutic treatment strategies, accurate viral load quantification is essential. Typically, in immunocompromised hosts, multiple viruses can reactivate simultaneously, which makes comprehensive identification of replicating pathogenic viruses essential. Currently, the monitoring of opportunistic viral infections in transplant patients is most frequently performed by multiple single-plex quantitative PCRs.

Metagenomic next-generation sequencing (mNGS) is increasingly being applied for the identification of pathogens in undiagnosed cases suspected of infection [2,3,4]. Quantification of viral loads utilising mNGS remains a challenge [5,6,7,8]. Complicating factors are the varying amount of background sequences from the host and from bacterial origin, technical bias affecting target sequence depth, unselective attribution of reads, and the number of calibration curves that are needed simultaneously when using untargeted sequencing for viral load calculations. Reports comparing mNGS with qPCR demonstrated a correlation with normalised sequence read counts but never as accurate as qPCR for viral load prediction [5]. Other previous research concerning the quantification of shotgun sequence read counts focused mainly on differential expression of RNA [9,10,11,12].

Recently, the Galileo Viral Panel (Arc Bio, LLC, Cambridge, MA, USA) has been designed as a quantitative mNGS approach for ten transplant-related DNA viruses [13,14]. This all-inclusive approach encompasses the library preparation kit, controls, calibration reagents, and cloud-based user-friendly software for bioinformatic analysis. Previous data on the performance of this mNGS approach demonstrated that the analytical performance was comparable to qPCR results with regard to the limits of detection, limits of quantification, and inter-assay variation [13,14]. 

In this study, we analysed the performance of the Galileo Viral Panel for viral load quantification in transplant patients over time. Subsequent samples from six transplant patients with proven infections or reactivations with transplantation-related DNA viruses (adenovirus, ADV; BK polyomavirus, BKV; cytomegalovirus, CMV; Epstein-Barr virus, EBV; human herpesvirus type 6A, HHV-6A; human herpesvirus type 6B, HHV-6B; herpes simplex type 1, HSV-1; herpes simplex type 2, HSV-2; JC polyomavirus, JCV; varicella-zoster virus, VZV; parvovirus B19, B19V; and torque teno virus, TTV) were analysed in comparison with qPCR. Accuracy of viral load quantification by mNGS was studied in relation to thresholds that had been used for the initiation of treatment or tapering of immunosuppression. Furthermore, we investigated the additional detection of DNA viruses identified by the broad mNGS approach, for which no targeted qPCR had initially been ordered.

## 2. Methods 

### 2.1. Patients and Sample Selection

Six adult immunocompromised patients (one allogeneic stem cell transplant patient, four kidney transplant patients, and one patient with hematological malignancy) were retrospectively selected based on available follow-up EDTA plasma samples that previously tested positive for one or more transplantation-related DNA viruses. Samples had previously (July 2008–December 2019) been sent to the Clinical Microbiological Laboratory (CML) of the Leiden University Medical Center (LUMC, The Netherlands) for viral load monitoring as part of routine patient care. Routine patient diagnostics consisted of several collection points, resulting in positive qPCR’s with a wide range of viral loads. CMV/EBV were routinely screened for in plasma post transplantation. BKV was screened in urine post renal transplantation; when positive it was also screened for in plasma. ADV and B19V were not routinely screened for but ordered at the discretion of the treating physician based on symptomatology. TTV viral load had been tested retrospectively by qPCR in the context of a different study. Patient plasma samples were stored at −80 °C until mNGS analysis.

### 2.2. Ethical Approval

Approval was obtained from the ethical committee from the LUMC (P11.165 NL 37682.058.11, and Biobank Infectious Diseases protocol 2020-03 & 2020-04 B20.002).

### 2.3. Extraction of Nucleic Acids; Internal Controls

Patient plasma samples were spiked with an internal control (baculovirus, Arc Bio, LLC) before extraction. Nucleic acids were extracted from 200 μL plasma using the MagNApure 96 DNA and Viral NA Small volume extraction kit on the MagNAPure 96 system (Roche Diagnostics, Almere, The Netherlands) with 100 μL output eluate. The eluate was concentrated using vacuum centrifugation by a SpeedVac vacuum concentrator (Thermo Scientific, Waltham, MA, USA) to a volume of 26 µL.

### 2.4. Library Preparation and Sequencing

Sequence libraries were prepared using the Galileo Viral Panel sequencing kit (Arc Bio, LLC, Cambridge, MA, USA) according to the manufacturer’s instructions. The protocol was based on enzymatic fragmentation at 37 °C for 5 min, followed by end repair and A-tailing at 65 °C for 30 min. Subsequently, fragments were ligated using unique dual-index adapters (ArcBio) at 20 °C for 15 min and purified using magnetic Kapa Pure Beads (Roche, Basel, Switzerland). No RNase treatment was included in the procedure, and human DNA was depleted using human depletion reagents at 45 °C for 2 h followed by 45 °C for 15 min, after which libraries were amplified using library amplification primers for 45 °C for 30 s, by 14 cycles of 98 °C for 10 s and 65 °C for 75 s and 65 °C for 5 min. The final library preparation products were purified using magnetic Kapa Pure Beads (Roche) and quantified using a Qubit fluorometer (Thermo Fisher, Waltham, MA, USA) followed by equally pooling using the Arc Bio calculation pooling tool. After a final quantity and quality check using a Bioanalyser (Agilent, Santa Clara, CA, USA), samples were sequenced using the NovaSeq 6000 sequencing system (Illumina, San Diego, CA, USA) at GenomeScan B.V. (Leiden, The Netherlands). For sequencing, S4 flowcells were used and samples were sequenced in two runs, where each pool consisted of around 12% of the lane capacity. Ten million reads per library were aimed for; the total reads per sample can be found in Table S1.

### 2.5. Calibration Samples

Initial calibration runs were performed testing the multi-analyte mixture (MAM) of whole-virus particles at viral loads of 0, 1000, 5000, 10,000, and 100,000 copies/mL or IU/mL plasma, in quintuple (Arc Bio, LLC) for the following 10 viruses: hADV-C1, BKV, CMV, EBV, HHV-6A, HHV6B, HSV-1, HSV-2, JCV, and VZV. For TTV and B19V, no Arc Bio calibrator panels were available, and therefore the Galileo Signal values were plotted against the calibrator plot of other viruses that demonstrated optimal agreement with the viral load (JCV and VZV, respectively), representing a semi-quantitative result.

### 2.6. Bioinformatic Analysis

After demultiplexing of the sequence reads using bcl2fastq (version 2.2.0) (Illumina, San Diego, CA, USA), FASTQ files were uploaded to the Galileo Analytics web application [13,15] which automatically processes data for quality assessment and pathogen detection using a custom database of DNA viruses involved in transplant-associated infections: ADV, CMV, EBV, HHV-6A, HHV-6B, HSV-1, HSV-2, JCV, VZV, B19V, and TTV. Human reads were removed before uploading the fastq files to the web application after mapping them to the human reference genome GRCh38 with Bowtie2 version 2.3.4 [6]. The analytics web application aligns sequence reads to the genomes of the DNA viruses in their calibration kit, scores these read alignments based on complexity, uniqueness, and alignment scores, and reports this in a signal value. The signal value is normalised for read counts across libraries, correcting for differences in genome lengths and technical bias, based on the spiked-in normalisation controls. The signals reported are related to the genomic depth and the observed amount of viral DNA being present in a sample, belonging to non-confounding genomic regions [13]. The sample signals were visualised in linear calibration curves (Figure S1).

### 2.7. Analysis of Performance and Additional Findings

Performance of the metagenomic Galileo Viral Panel assay was assessed in comparison with routine qPCR, analysing both qualitative and quantitative detection. Additional findings by mNGS were confirmed by additional qPCR analysis. In case no remaining sample was available, the Galileo Analytics software results were compared with results from the analysis using alternative bioinformatic tools: metagenomic taxonomic classifier Centrifuge (1.0.4-beta) [16] and de novo assembly-based viral metagenomic analysis software Genome Detective [17]. 

## 3. Results

### 3.1. Calibration Curves

After metagenomic sequencing, the viral loads were calculated for each virus by the Galileo Analytics web application. Signals of both the calibrators and patient plasma samples were plotted in load graphs (Figure S1) and the corresponding viral load of the patient samples was extrapolated. As no calibrator panels for B19V and TTV virus were available, these signals were plotted against other calibration curves of viruses that demonstrated the optimal agreement with the known viral load for semi-quantitative detection. All calibration sample signals correlated well with the titre (R^2^ range 0.84–0.92). 

### 3.2. Viral Load by mNGS Versus qPCR

**Figure 1:**
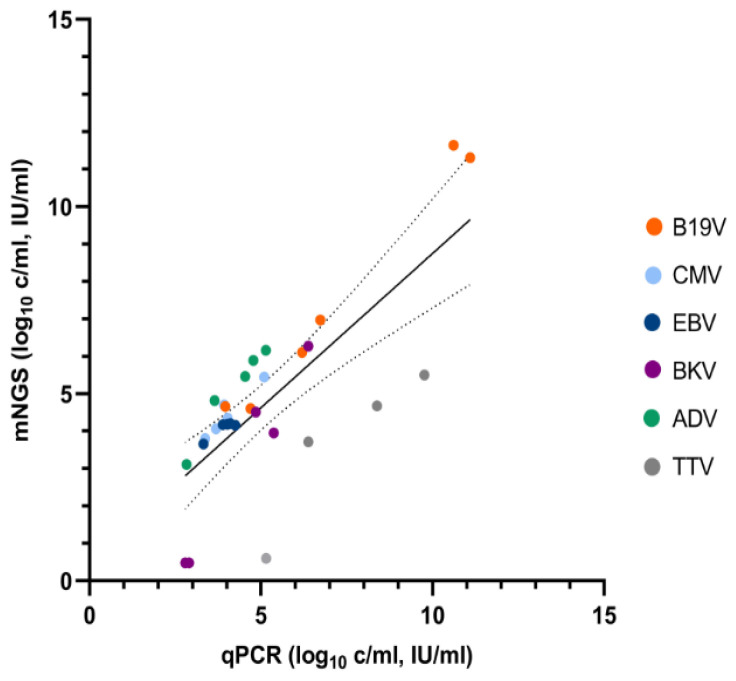
Viral loads as predicted by Galileo Viral Panel mNGS versus qPCR (copies/mL for ADV, BK, and TTV, and IU/mL for CMV, EBV, and B19V). B19V and TTV results were considered semi-quantitative, as no Galileo calibration panels were available for these targets.

In total, six patients were tested by qPCR and mNGS for quantification of different viruses at subsequent time points. The agreement between the methods for qualitative detection was 100% for the viruses targeted by PCR. Quantitative results per patient are shown in Table 1, and Figure 1 depicts viral loads by mNGS versus qPCR per target virus. CMV and EBV viral loads demonstrated the highest agreement, with a maximum difference in viral load of 0.70 log_10_ IU/mL. Mean differences in viral loads were 0.43 for CMV and 0.19 log_10_ IU/mL for EBV. Genotyping had not been performed for ADV (patient 1) and TTV (patient 4) in the context of routine care but resulted in the human adenovirus 1 and TTV-like mini virus, respectively, using mNGS data (based on de novo genome assembly followed by blastn). Viral loads were higher when quantified with mNGS with a mean difference of 0.90 log_10_ c/mL. For BKV, viral loads by mNGS were lower in comparison with qPCR, with a mean difference of 1.32 log_10_ c/mL. When taking into account viral loads measured above the limit of quantification of 2.5 log_10_ c/mL, as applied in our diagnostic qPCR for BKV, the mean difference is 0.62 log_10_ c/mL and a trend towards a better agreement with higher viral loads could be observed. Semi-quantitative detection of B19V and TTV viruses by mNGS resulted in mean differences of, respectively, 0.39 log_10_ IU/mL and 3.0 log_10_ c/mL in comparison with qPCR. 

### 3.3. Longitudinal Patient Follow-Up and Clinical Decision Making

**Figure 2:**
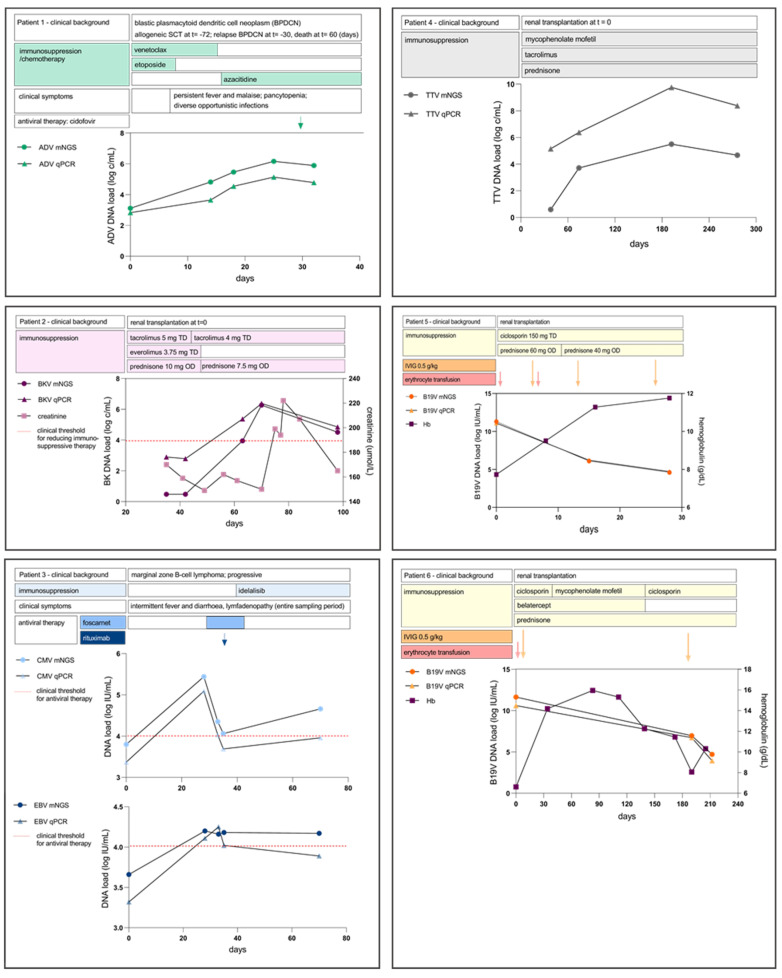
Longitudinal follow-up of DNA viral loads in immunosuppressed patients over time, as predicted by mNGS (Galileo Viral Panel, Arc Bio) versus qPCR. Clinical information and therapeutic agents are included.

Table 2 gives an outline of patient characteristics and provides clinical information on underlying conditions and complications during the sampling period. Furthermore, for each patient, the viral loads over time were plotted in graphs with clinical information, symptomatology, relevant laboratory parameters, and treatment (Figure 2). For CMV, EBV, and BKV, in our clinical practice, specific viral load thresholds are used to decide whether immunosuppression should be tapered and/or antiviral therapy should be administered. Viral load quantification around these thresholds demonstrated good agreement in identifying these clinical decision-making breakpoints. In Patient 3, the antiviral treatment with Foscarnet was started for CMV-reactivation when viral load measured by qPCR exceeded 4.0 log_10_ IU/mL. By mNGS, this critical threshold for treatment initiation was correctly identified with a viral load by mNGS of 5.44 log_10_ IU/mL. In the same patient, rituximab was administered when the EBV load by qPCR was repeatedly above the threshold of 4.0 log_10_ IU/mL, consistently quantified thrice above 4.0 log_10_ IU/mL before administration of rituximab, both by qPCR and mNGS. 

For B19V, ADV, and TTV, no predefined thresholds were used for changing the treatment regimen. For all viruses, the observed trends in load over time in each patient were comparable for qPCR and mNGS, despite the semi-quantitative nature of the B19V mNGS assay. Effect of treatment (anti-viral drugs, immunoglobulins, and/or tapering of immunosuppressive drugs) in patients was estimated by follow-up of viral loads by qPCR. For B19V in Patients 5 and 6, the effect of intravenous immunoglobulins (IVIG) could be assessed by the decreasing viral load in the weeks after administration, as also observed by mNGS. For ADV, in patient 1, antiviral therapy with cidofovir was started when a consistent increase in viral load was detected, both by qPCR and mNGS.

### 3.4. Additional Findings

For some samples, additional viral reads were detected in the pathogenic mNGS reports that were not initially tested for by qPCR (Table S1). Most additional findings were supported by a secondary bioinformatic analysis using the Centrifuge and Genome Detective: BK (1 patient), CMV (1 patient), HHV-6B (1 patient), and TTV (4 patients, torque teno virus was the deepest level of classification obtained, using mNGS data, with lower than 100% genome coverage). In a few cases, additional findings were not confirmed by a second analysis, leaving some low mNGS signals for CMV, EBV, and HSV. JCV was detected by mNGS in a sample with a high concentration of BKV, which possibly indicated forced alignment contamination due to high sequence homology between JCV and BKV [13,14]. 

## 4. Discussion

In this study, the performance of a quantitative mNGS assay for the longitudinal follow-up of DNA viral loads was analysed in six immunocompromised patients. Viral loads determined by mNGS were comparable with loads determined by qPCR, and differed less than 1 log_10_ for DNA viruses with calibration panels available, in line with previous studies [13,14]. In the current study, the performance of viral loads assessed by mNGS was also evaluated with regard to clinical decision making. In the management of reactivating viruses in immunocompromised patients, local and international guidelines use viral load breakpoints to decide whether antiviral therapy should be administered or whether immunosuppression should be tapered [18,19,20,21,22]. Viral loads under investigation in this study were determined by qPCR as part of routine patient care. When local clinical breakpoints were considered for each virus, mNGS performed comparably to qPCR to identify the clinically relevant breakpoints. B19V is not considered to be a reactivating virus, but quantification may be helpful to distinguish clinically relevant replicative infection from merely DNA remnants [23]. In the range of these breakpoints, viral loads were adequately determined by mNGS to guide clinical decision making. Additionally, the longitudinal trend was similar in comparison with qPCR, indicating precision of mNGS for clinical quantification and reliable indication of the trend in viral load. Clinical decision making is often guided by follow-up of viral load trends, in addition to the cross-sectional viral load measurements for viral infections without available thresholds. In the future, more research is desired to analyse the performance in the lower ranges to map the limit of quantification (LOQ) of mNGS procedures. It is anticipated that the LOQ is somewhat higher than the LOQ of qPCR, given the generally higher limit of detection in combination with the variability of mNGS, mainly resulting from the varying amounts of background sequences. 

The principle of a quantitative catchall approach to detect all transplantation-related viruses in a single run is an attractive feature in the clinical follow-up of the immunocompromised host. Simultaneous reactivation of persistent viruses during immunocompromised episodes is common. Co-infection rates of up to 32% have been described using PCR and, importantly, were associated with higher rates of acute rejection or graft dysfunction [24]. Co-infections may be missed when ordering targeted PCRs, while the catchall approach of mNGS could guarantee that active infections are not overlooked. Indeed, our approach demonstrated a complementary yield of seven reactivating viruses in five patients, which had not been identified earlier by qPCR. Some of these unnoticed viruses are not considered pathogenic, such as TTV. However, the role of TTV in clinical management is still developing, as recent and ongoing research suggests its potential as marker of functional immunity, with an inverse correlation between TTV-load and risk of rejection. Clinical trials exploring its role as a marker for balancing immunosuppressive treatment, with a focus on tacrolimus, are currently being conducted (e.g., ClinicalTrials.gov NCT04198506) [25,26,27,28]. ADV, generally, is not systematically screened for in the severely immunosuppressed adult population. In our patient, although actively diagnosed, ADV-loads were rapidly increasing and a catchall approach could guarantee that such less common infections are not overlooked, especially in the absence of localizing symptoms.

A significant complementary virus identification yield by mNGS in transplant patients of 31/49 plasma samples was also reported by Sam et al. [14], with the majority, being viruses, considered pathogenic. These findings demonstrate that mNGS could improve pathogen detection in clinical practice.

Another advantage of mNGS would be its capacity to genotype viruses and detect mutations associated with antiviral resistance, without the need for additional, time-consuming, target-specific ‘wet’ lab procedures that could delay diagnosis and treatment. As an example, Patient 3 in our study was treated with Foscarnet for persistent CMV reactivation pending the results of mutational analysis after clinical failure of valganciclovir treatment. If the results of mutational analysis had been immediately available, resorting to second-line treatment may have been avoided.

Widespread implementation of mNGS approaches in clinical diagnostic settings has been limited by several factors. The ‘wet’ lab protocols can be time-consuming, costly, and have a relatively long turnaround time, mainly due to the time required for sequencing. With various sequencing techniques still rapidly evolving, the costs and sequencing turnaround time of such protocols are expected to improve considerably in the future [29]. Furthermore, bioinformatic skills are generally needed for validation and implementation as a diagnostic assay. User-friendly, all-in-one mNGS data analysis software packages for cloud-based and automated analysis enable use in laboratories with minimal bioinformatic knowledge and allow access to high-performance computing capacity.

Limitations in this current study are the relatively low number of samples and viruses when considering a metagenomic approach, including two viruses without calibration panels available. This small-scale study provides a proof-of-principle demonstration in a retrospective design demonstrating that the current version of the Research Use Only Galileo Viral Panel enables longitudinal viral load monitoring by mNGS. It is expected that, after these initial studies, indicating high performance in terms of limit of detection and quantification, inter-run precision, and prospective viral load monitoring, the kit and software will be expanded to include more viruses, calibration samples, and potentially fit for different sample types. Furthermore, technical and bioinformatic features might be evolved in future versions of the assay.

Overall, viral metagenomic sequencing is a promising approach not only for DNA virus detection and identification, but also for reliable estimation of the viral load in a clinical setting, and potentially mutational typing for drug sensitivity analysis. Several milestones essential for implementation in diagnostic settings have been met by the specific assay used in this study: the limits of detection, the limits of quantification, precision, and overall technical performance, which were comparable with qPCR assays. Precise quantification was accomplished by read normalisation based on a designed control. These accomplishments pave the way for further developments and optimisation of quantitative metagenomic sequencing for longitudinal viral load monitoring and beyond.

## Figures and Tables

**Table 1:** Viral load quantification by qPCR and mNGS per patient sample.

Patient-Sample	Virus	Viral Load qPCR	Viral Load qPCR (log_10_)	Viral Load mNGS	Viral Load mNGS (log_10_)	ΔqPCR-mNGS (log_10_)
P1-S1	ADV	675 c/mL	2.83 c/mL	1277 c/mL	3.11 c/mL	0.28 c/mL
P1-S2		4517	3.65	66,273	4.82	1.17
P1-S3		34,740	4.54	287,844	5.46	0.92
P1-S4		136,900	5.14	1,435,130	6.16	1.02
P1-S5		60,540	4.78	777,172	5.89	1.11
P2-S1	BKV	796 c/mL	2.90 c/mL	3 c/mL	0.48 c/mL	−2.42 c/mL
P2-S2		614	2.79	3	0.48	−2.31
P2-S3		233,700	5.37	9011	3.95	−1.41
P2-S4		2,401,000	6.38	1,857,785	6.27	−0.11
P2-S5		71,480	4.85	32,321	4.51	−0.34
P3-S1	CMV	2370 IU/mL	3.37 IU/mL	6,246 IU/mL	3.80 IU/mL	0.42 IU/mL
P3-S2		122,800	5.09	275,657	5.44	0.35
P3-S3		10,680	4.03	22,242	4.35	0.32
P3-S4		4915	3.69	11,366	4.06	0.36
P3-S5		9156	3.96	46,231	4.66	0.70
P3-S1	EBV	2083 IU/mL	3.32 IU/mL	4581 IU/mL	3.66 IU/mL	0.34 IU/mL
P3-S2		12,970	4.11	1573	4.20	0.09
P3-S3		17,710	4.25	14,549	4.16	−0.09
P3-S4		10,500	4.02	15,077	4.18	0.16
P3-S5		7723	3.89	14,844	4.17	0.28
P4-S1	TTV *	140 c/mL	2.15 c/mL	4 c/mL	0.60 c/mL	−1.54 c/mL
P4-S2		2,400,000	6.38	5142	3.71	−2.67
P4-S3		5.7 × 10^9^	9.76	319,074	5.50	−4.25
P4-S4		2.4 × 10^8^	8.38	46,261	4.67	−3.71
P5-S1	B19V *	1.34 × 10^11^ IU/mL	11.13 IU/mL	2.07 × 10^11^ IU/mL	11.32 IU/mL	0.19 IU/mL
P5-S2		1,407,365	6.15	1,235,416	6.09	−0.06
P5-S3		45846	4.66	41,787	4.62	−0.04
P6-S1	B19V *	4.07 × 10^10^ IU/mL	10.61 IU/mL	4.37 × 10^11^ IU/mL	11.64 IU/mL	1.03 IU/mL
P6-S2		5,309,308	6.73	9,376,953	6.97	0.25
P6-S3		8569	3.93	49,601	4.70	0.76

* B19V and TTV results were considered semi-quantitative, as no Arc Bio calibration samples were available for these targets.

**Table 2:** Patient characteristics and clinical background at start of longitudinal follow-up.

Patient Number	Virus	Age Range	Sex	Underlying Condition	Conditioning Regimen	Transplantation	Other Known Infectious Complications During Sampling Period
1	ADV	60–79	V	Blastic plasmacytoid dendritic cell neoplasm (BPDCN)	Two failed remission-induction regimens; followed by t * = −3: COPADM † t= −2: COPADM	t = 0: Non-myeloablative allogeneic stem cell transplant from unrelated donor; t = 1: relapse BPDCN	1. Probable pulmonal aspergillosis 2. CMV reactivation treated with foscarnet (week before sampling period) 3. *Enterococcus faecalis* UTI ‡
2	BKV	20–39	M	Chronic renal insufficiency due to TIN ¥, as an extraintestinal manifestation of known colitis ulcerosa or medicine-induced	Alemtuzumab	Pre-emptive living-related renal transplant	1. CMV reactivation
3	CMV, EBV	60–79	V	Marginal zone B-cell lymphoma; established 4 years previously, now progressive	Recent chemotherapy: t = −6: CHOP ¦	Not applicable	1. *Escherichia coli* UTI 2. rhinovirus RTI
4	TTV	40–59	V	IgA nephropathy	Basiliximab	Living-related renal transplant	1. *Escherichia coli* UTI
5	B19V	40–59	M	IgA nephropathy	Basiliximab	pre-emptive living-unrelated renal transplant	
6	B19V	40–59	M	Focal segmental glomerulosclerosis (FSGS)	Not applicable	Non-heart beating renal transplant 4 years previously; 15 years previously living-related renal transplant	

* t = time in months; † COPADM = cyclophosphamide, oncovin (vincristine), prednisone, Adriamycin (doxorubicin), methotrexate; ‡ UTI = urinary tract infection; ¥ TIN = tubulointerstitial nefritis; ¦ CHOP = cyclophosphamide, oncovin (vincristine), Adriamycin, prednisone; ¶ RTI = respiratory tract infection. For a complementary longitudinal overview of symptomatology, including laboratory parameters and treatment, see Figure 2.

## Data Availability

Not applicable.

## Supplementary Materials

The following are available online at https://www.mdpi.com/article/10.3390/pathogens11020236/s1, Figure S1: Calibration graphs of the six viruses in six patients in this study with associated slope, intercepts and R2 values; Table S1: Additional findings of the metagenomic Galileo Viral Panel compared to Centrifuge and Genome Detective software.
