# Supplementary material for: Longitudinal Monitoring of DNA Viral Loads in Transplant Patients Using Quantitative Metagenomic Next-Generation Sequencing
Source: Pathogens. 2022 Feb 11;11(2):236. doi: 10.3390/pathogens11020236 (PMC8874692; doi:10.3390/pathogens11020236)
Supplement: Supplementary file 1 [file pathogens-11-00236-s001.zip › NEW_Supplementary_Figure S1.pdf]

Supplementary Figure S1. Calibration graphs of the six viruses in six patients in this study with associated slope, intercepts and R^2 values. Concentrations are expressed in $\log_{10}$ copies or IU/ml. Calibrator samples are shown in black dots, clinical samples in orange. **A**: ADV; slope = 0.92, intercept = 0.33, R^2 = 0.92; **B**: B19V; slope = 1.01, intercept = 0.45, R^2 = 0.91; **C**: CMV; slope = 0.9, intercept = 0.18, R^2 = 0.89; **D**: EBV; slope = 0.97, intercept = 0.04, R^2 = 0.9; **E**: BKV; slope = 0.98, intercept = 0.42, R^2 = 0.92; **F**: TTV; slope = 0.81, intercept = 0.57, R^2 = 0.84

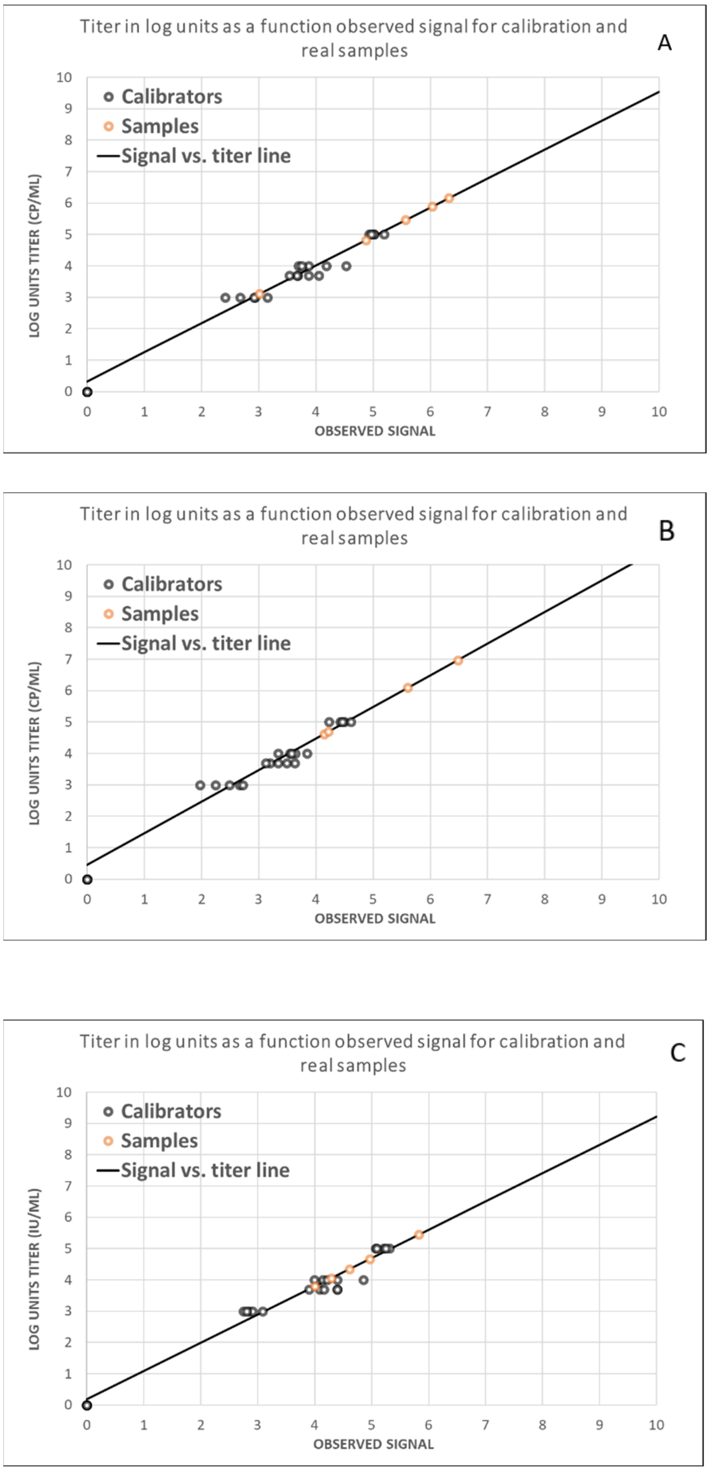

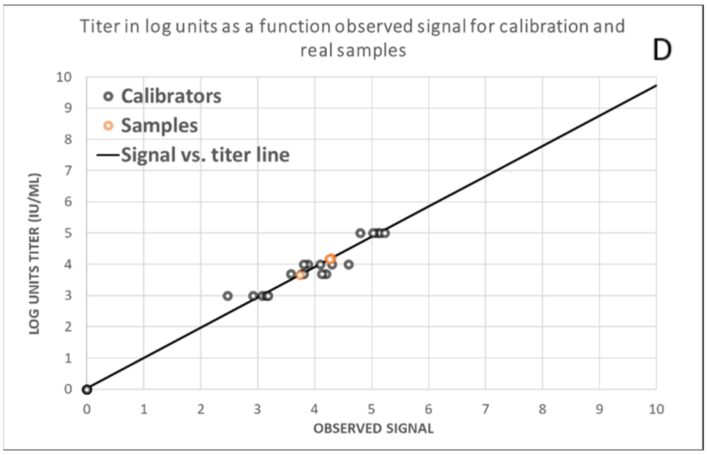
Titer in log units as a function observed signal for calibration and real samples
D
Calibrators
Samples
Signal vs. titer line
LOG UNITS TITER (IU/ML)
OBSERVED SIGNAL
0 1 2 3 4 5 6 7 8 9 10

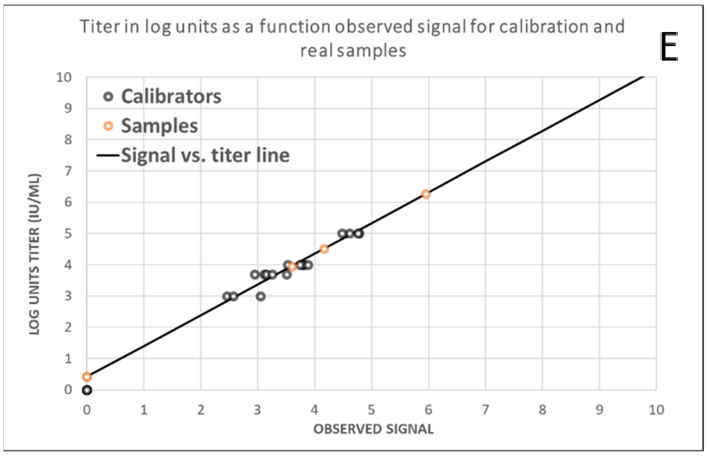
Titer in log units as a function observed signal for calibration and real samples
E
Calibrators
Samples
Signal vs. titer line
LOG UNITS TITER (IU/ML)
OBSERVED SIGNAL
0 1 2 3 4 5 6 7 8 9 10

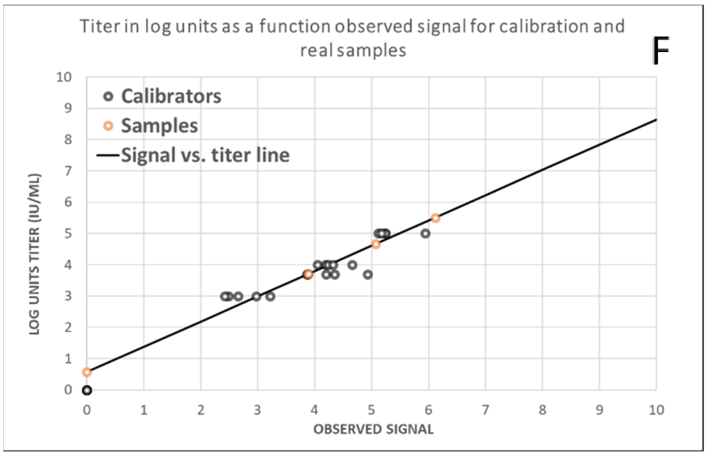
Titer in log units as a function observed signal for calibration and real samples
F
Calibrators
Samples
Signal vs. titer line
LOG UNITS TITER (IU/ML)
OBSERVED SIGNAL
0 1 2 3 4 5 6 7 8 9 10
