# Supplementary material for: Longitudinal Monitoring of DNA Viral Loads in Transplant Patients Using Quantitative Metagenomic Next-Generation Sequencing
Source: Pathogens. 2022 Feb 11;11(2):236. doi: 10.3390/pathogens11020236 (PMC8874692; doi:10.3390/pathogens11020236)
Supplement: Supplementary file 1 [file pathogens-11-00236-s001.zip › NEW_Supplementary_Table S1_09-02.pdf]

Supplementary Table S1. Additional findings of the metagenomic Galileo Viral Panel compared to Centrifuge and Genome Detective software, including total read counts. For Galileo Analytics, results are presented as viral load in log_{10} c/mL or IU/ml (see also table 1). For Centrifuge and Genome Detective, results are presented as absolute amount of reads classified per species or genus. For TTV reads during centrifuge analysis, Anelloviridae reads are shown. * Limited sample being available for additional testing. All additional mNGS Galileo Analytics findings that were tested for qPCR were found positive.

P1 ADV		S1	S2	S3	S4	S5
Total read counts		21005194	16547546	30828990	18318968	1961070
BKV	Galileo Analytics		3.67	4.03*	3.78*	
	Centrifuge		47	74	67	
	Genome Detective		84	113	35	
CMV	Galileo Analytics	2.45*	3.69*	3.84*	3.84*	3.16*
	Centrifuge	18	123	83	53	1
	Genome Detective	0	12	156	2	0
EBV	Galileo Analytics		1.22			
	Centrifuge		0			
	Genome Detective		0			
B19V	Galileo Analytics	3.85	3.65		3.55	
	Centrifuge	0 (1 AADPA†)	2 (147 AADPA)		0 (1 AADPA)	
	Genome Detective	0	(140 AAV2‡)		(124 AAV2)	
TTV	Galileo Analytics	5.54	6.18	4.98	4.61	4.39
	Centrifuge	2382	3525	489	137	2
	Genome Detective	11426	22595	2719	823	2
P2 BKV		S1	S2	S3	S4	S5
Total read counts	532064	6276164	7340891	15404463	5446384	
ADV	Galileo Analytics	2.94		2.94		2.44
	Centrifuge	3		0		1
	Genome Detective	0		0		0

CMV	Galileo Analytics		2.6*	4.46*	5.07*	
	Centrifuge		13	279	1056	
	Genome Detective			110	975	
TTV	Galileo Analytics	3.87	4.84	5.54	4.71	4.99
	Centrifuge	96	1196	953	56	1511
	Genome Detective	406	7037	5900	241	11141
VZV	Galileo Analytics			2.3		
	Centrifuge			1		
	Genome Detective			0		
JCV	Galileo Analytics				4.06	
	Centrifuge				0	
	Genome Detective				0	
HSV	Galileo Analytics		0.87			
	Centrifuge		1			
	Genome Detective					
P3 CMV/EBV		S1	S2	S3	S4	S5
Total read counts	6931650	13589398	16713704	24942480	11789406	
BKV	Galileo Analytics		4.99	4.98	4.89	5.12
	Centrifuge		22	60	33	21
	Genome Detective		0	38	4	0
B19V	Galileo Analytics	4.69	4.93	5.52	5.16	5.17
	Centrifuge	0	1	0	1	2
	Genome Detective	0	0	0	0	0
TTV	Galileo Analytics	5.21		5.19	5.25	5.66
	Centrifuge	22		45	35	131
	Genome Detective	37		168	97	547
HHV6A	Galileo Analytics		3.48			

	Centrifuge		1			
	Genome Detective		0			
HHV6B	Galileo Analytics		4.11			
	Centrifuge		14			
	Genome Detective		14			
P4 TTV		S1	S2	S3	S4	
Total read counts	15398234	15371204	31506646	13252084		
CMV	Galileo Analytics	2.95	3.46			
	Centrifuge	26	38			
	Genome Detective	28	53			
B19V	Galileo Analytics	3.98	4.16	4.07	4.27	
	Centrifuge	2	1	1	3	
	Genome Detective	0	0	0	0	
P5 B19V		S1	S2	S3		
Total reads	32130834	11167894	14742234			
CMV	Galileo Analytics	2.71		3.46*		
	Centrifuge	2		27		
	Genome Detective	4		52		
EBV	Galileo Analytics		2.08			
	Centrifuge		0			
	Genome Detective		0			
BKV	Galileo Analytics			3.81*		
	Centrifuge			12		
	Genome Detective			20		
HSV1	Galileo Analytics			3.25		
	Centrifuge			12		
	Genome Detective			0		
P6 B19V		S1	S2	S3		

Total reads	24530794	16340614	19282400			
ADV	Galileo Analytics			2.72		
	Centrifuge			1		
	Genome Detective			0		
EBV	Galileo Analytics		1.19			
	Centrifuge		0			
	Genome Detective		0			
TTV	Galileo Analytics	6.58	2.83	2.70		
	Centrifuge	1681	10094	3		
	Genome Detective	10100	17873	0		

† AADPA = Adeno-associated dependoparvovirus A; ‡AAV2 = adeno-associated virus 2
